# Dihydropyrimidone Derivatives as Thymidine Phosphorylase Inhibitors: Inhibition Kinetics, Cytotoxicity, and Molecular Docking

**DOI:** 10.3390/molecules28083634

**Published:** 2023-04-21

**Authors:** Tian-Meng Cui, Muhammad Altaf, Abdu Aldarhami, Abdulrahman S. Bazaid, Nizar H. Saeedi, Almohanad A. Alkayyal, Fahad M. Alshabrmi, Farman Ali, Mohammed Aladhadh, Muhammad Yasir Khan, Ahad Amer Alsaiari, Yue-Rong Ma

**Affiliations:** 1 School of Basic Medicine, Chengdu University of Traditional Chinese Medicine, Chengdu 610075, China; 2 Department of Biochemistry, Federal Urdu University of Arts, Sciences and Technology, Karachi 75300, Pakistan; 3 Department of Medical Microbiology, Qunfudah Faculty of Medicine, Umm Al-Qura University, Al-Qunfudah 21961, Saudi Arabia; 4 Department of Medical Laboratory Science, College of Applied Medical Sciences, University of Ha’il, Hail 55476, Saudi Arabia; 5 Department of Medical Laboratory Technology, Faculty of Applied Medical Sciences, University of Tabuk, Tabuk 71491, Saudi Arabia; 6 Department of Medical Laboratories, College of Applied Medical Sciences, Qassim University, Buraydah 51452, Saudi Arabia; 7 Department of Chemistry, Federal Urdu University of Arts, Sciences and Technology, Karachi 75300, Pakistan; 8 Department of Food Science and Human Nutrition, College of Agriculture and Veterinary Medicine, Qassim University, Buraydah 51452, Saudi Arabia; 9 Department of Biology, Faculty of Science, King Abdulaziz University, Jeddah 21589, Saudi Arabia; 10 Vaccine and Immunotherapy Unit, King Fahad Medical Research Center, King Abdulaziz University, Jeddah 21589, Saudi Arabia; 11 Department of Clinical Laboratory Sciences, College of Applied Medical Sciences, Taif University, Taif 21944, Saudi Arabia

**Keywords:** thymidine phosphorylase (TP), angiogenesis, dihydropyrimidone derivatives, non-competitive inhibition, molecular docking, anti-cancer

## Abstract

Overexpression of the thymidine phosphorylase (TP) enzyme induces angiogenesis, which eventually leads to metastasis and tumor growth. The crucial role of TP in cancer development makes it an important target for anticancer drug discovery. Currently, there is only one US-FDA-approved drug, i.e., Lonsurf, a combination of trifluridine and tipiracil, for the treatment of metastatic colorectal cancer. Unfortunately, numerous adverse effects are associated with its use, such as myelosuppression, anemia, and neutropenia. Since the last few decades, the discovery of new, safe, and effective TP inhibitory agents has been rigorously pursued. In the present study, we evaluated a series of previously synthesized dihydropyrimidone derivatives **1**–**40** for their TP inhibitory potential. Compounds **1**, **12**, and **33** showed a good activity with IC_50_ = 314.0 ± 0.90, 303.5 ± 0.40, and 322.6 ± 1.60 µM, respectively. The results of mechanistic studies revealed that compounds **1**, **12**, and **33** were the non-competitive inhibitors. These compounds were also evaluated for cytotoxicity against 3T3 (mouse fibroblast) cells and were found to be non-cytotoxic. Finally, the molecular docking suggested the plausible mechanism of non-competitive inhibition of TP. The current study thus identifies some dihydropyrimidone derivatives as potential inhibitors of TP, which can be further optimized as leads for cancer treatment.

## 1. Introduction

Angiogenesis is the process by which new blood vessels form from pre-existing ones. It is a crucial process in normal development and tissue repair, but it can also be exploited by cancer cells to support their growth and spread. Tumors require a blood supply to deliver oxygen and nutrients, and angiogenesis provides a means for this. Additionally, angiogenesis can allow cancer cells to escape from the primary tumor and travel to distant sites in the body to form new tumors. There has been significant interest in targeting angiogenesis as a strategy to treat cancer [1,2]. While anti-angiogenic therapies have shown promise in treating some types of cancer, there are still challenges to be addressed.

Thymidine phosphorylase (TP) is an enzyme that plays a crucial role in the process of angiogenesis, which is the formation of new blood vessels. TP is over-expressed in many types of cancer, and its expression is associated with tumor growth, invasion, and metastasis. TP catalyzes the breakdown of thymidine into the following two products: thymine and 2′-deoxy-D-ribose-1-phosphate. The latter product, 2′-deoxy-D-ribose-1-phosphate, is then dephosphorylated to produce 2′-deoxy-D-ribose [3]. This molecule can stimulate the production of vascular endothelial growth factor (VEGF), a protein that plays a critical role in angiogenesis. VEGF activates endothelial cells, which line the walls of blood vessels and promotes their proliferation and migration. This process is further facilitated by the secretion of matrix metalloproteinases (MMPs), which are enzymes that break down extracellular matrix proteins and facilitate the remodeling of tissues. Together, these processes lead to the formation of new blood vessels, which can supply nutrients and oxygen to cancer cells and facilitate their spread to other parts of the body [4].

TP is a highly conserved enzyme that has been found in a wide range of organisms, including bacteria, plants, and animals. The primary amino acid sequence of TP is generally well conserved across different species, suggesting that the enzyme has an important and conserved biological function. It has been reported that TP from mammalian sources, such as humans, has a high degree of sequence similarity with TP from other mammals. Interestingly, TP from mammalian sources also shares a sequence similarity with TP from bacteria, including Escherichia coli. The amino acid sequence of TP from E. coli has been reported to be up to 39% identical to that of TP from mammals [5]. The active site of mammalian TP enzyme also shares around 65–70% sequence similarity with the active site residues of E. coli TP enzyme [6].

Since TP inhibition modulates the formation of a key product, i.e., 2′-deoxy-D-ribose, which leads to tumor growth suppression [7]. TP inhibition is thus a vital approach against angiogenesis and cancer. Lonsurf (trifluridine and tipiracil) and 5-fluorouracil are currently the only FDA-approved drugs for the treatment of metastatic colorectal cancer that has progressed after standard therapies [8]. Like many cancer drugs, Lonsurf can cause side effects, and some of the common side effects of this drug include anemia, neutropenia, and myelosuppression. Other less common side effects may include fatigue, nausea, vomiting, and loss of appetite. Hence, there is an urgent need for novel, safe, and effective TP inhibitory agents [9]. A pyrimidine analog known as TPI (5-chloro-6-[1-(2-iminopyrrolidinyl) methyl] uracil hydrochloride) was identified as the most potent inhibitor of human TP till date. In addition to it, **7-deazaxanthine** (a purine analog) is a commonly used reference compound for in vitro TP inhibitory assay [10,11]. Previously, different analogs of triazines, i.e., 5-chlorouracil-linked-pyrazolo [1,5-α] [1,3,5] triazines, 1,2,4-triazolo [1,5-a] [1,3,5] triazin-5,7-dione, and 1,3,5-triazin-2,4-dione were described by different researchers for their inhibitory potential against TP enzyme [12,13,14]. Previously, the dihydropyrimidone derivatives also showed significant activity against TP [15]. Since the last decade, our research group has focused on the discovery of leads against thymidine phosphorylase. In continuation of our studies, we report here the TP inhibitory activity of a previously synthesized series of dihydropyrimidone derivatives [16].

Keeping in view the extensive medicinal importance of dihydropyrimidones, as well as their structural similarity with **7-deazaxanthine**, a class of dihydropyrimidones derivatives was synthesized and previously reported by our research group [16]. In this manuscript, we report the in vitro TP inhibitory activity of 40 different dihydropyrimidones derivatives. Potent compounds of the series were also subjected to cytotoxicity evaluation against the 3T3 (mouse fibroblast) cell line using MTT (3-[4,5-dimethylthiazole-2-yl]-2,5-diphenyl-tetrazolium bromide) spectrophotometric assay [17]. Kinetic studies were also performed to identify the mode of action of active compounds. The Kinetic studies revealed that the most active compounds were non-competitive inhibitors of TP. We also performed the molecular docking study to rationalize the mechanism of TP inhibition.

## 2. Results and Discussion

### 2.1. In Vitro Study

Differently substituted dihydropyrimidone derivatives **1**–**40** were screened; among them, twenty-one compounds exhibited some level of activity against the TP enzyme (showed more than 50% inhibition) (Table 1). Compound **12** (IC_50_ = 303.5 ± 0.42 µM), **1** (IC_50_ = 314.3 ± 0.93 µM), and **33** (IC_50_ = 322.6 ± 1.68 µM) showed a significant activity when compared with standard inhibitors of the study, i.e., **7-deazaxanthine** (IC_50_ = 41.0 ± 1.63 µM) and tipiracil-HCl (IC_50_ = 0.014 ± 0.04 µM).

**Figure 1:**
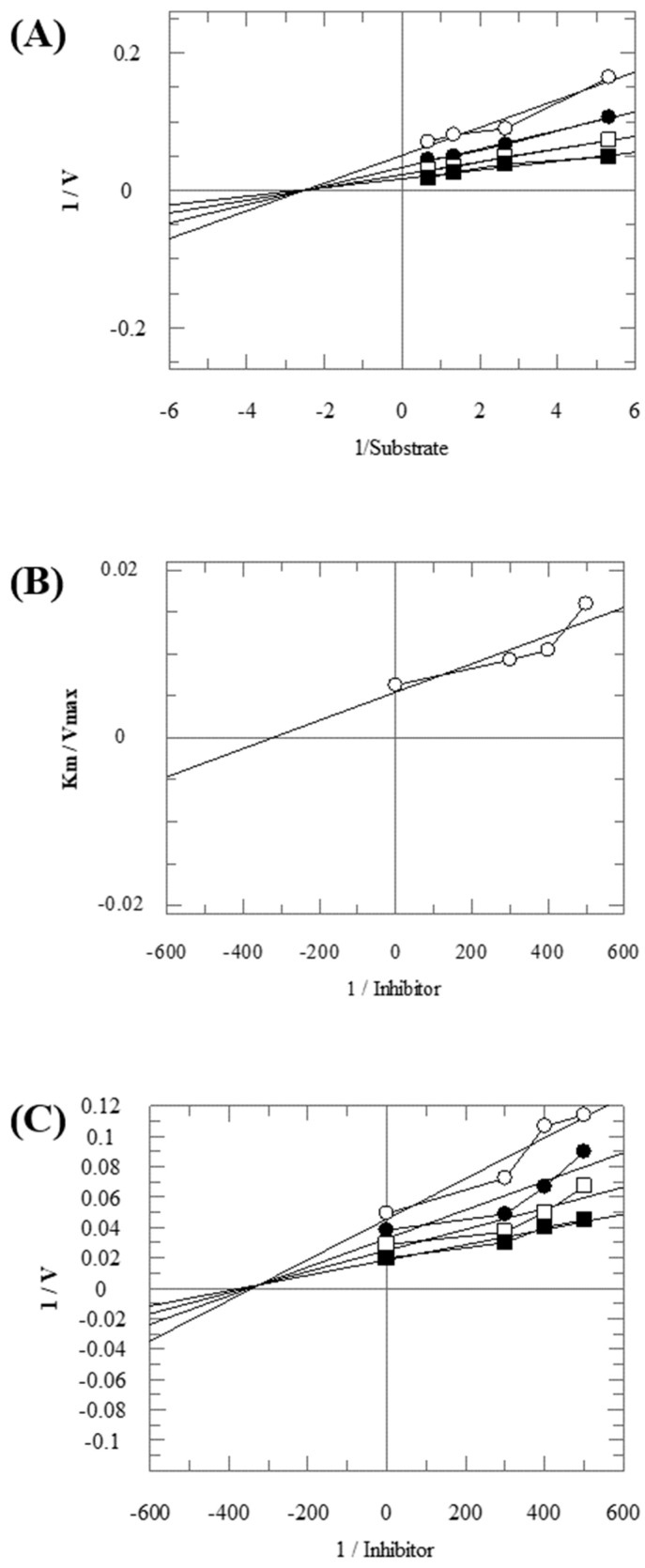
The inhibition of thymidine phosphorylase (TP) by compound **1**. (**A**) Lineweaver–Burk plot presented 1/V on their Y-axis while that of reciprocal of the substrate (thymidine) on their X-axis. Order of the concentrations of inhibitor included in the absence of compound **1** (■), in the presence of 300 µM (□), 400 µM (●), and 500 µM (○) of compound **1**. (**B**) Secondary replot of Lineweaver–Burk was plotted between the slopes (Km/Vmax) on Y-axis and 1/Inhibitor on X-axis, and (**C**) Dixon plot presented 1/V on Y-axis, while 1/Inhibitor on X-axis.

To understand the structure–activity relationship (SAR), we considered compound **1** as the parent compound (i.e., with unsubstituted benzene ring) on which different substitutions made resulting compounds active, weakly active, or inactive. Results revealed that compound **1** was the second most potent compound of the series with an IC_50_ value of 314.3 ± 0.9 µM; however, when we compared it with standard inhibitors of the study, i.e., tipiracil-HCl (IC_50_ = 0.014 ± 0.04 µM) and **7-deazaxanthine** (IC_50_ = 41.0 ± 1.63 µM), it was found to be weakly active and thus need structural optimization. To characterize the mechanism of enzyme inhibition, compound **1** was subjected to kinetic studies. Results revealed that compound **1** was a non-competitive type of inhibitor with Ki = 326.3 ± 0.002 µM (Table 2; Figure 1), while the dose–response curve of compound **1** with standard deviation is provided in the Supplementary File Figure S1. From these results, it is suggested that compound **1** might interact with non-active site amino acid residues of the enzyme and thus inhibit its activity. These interactions might be through hydrogen bonding or hydrophobic interactions.

**Figure 2:**
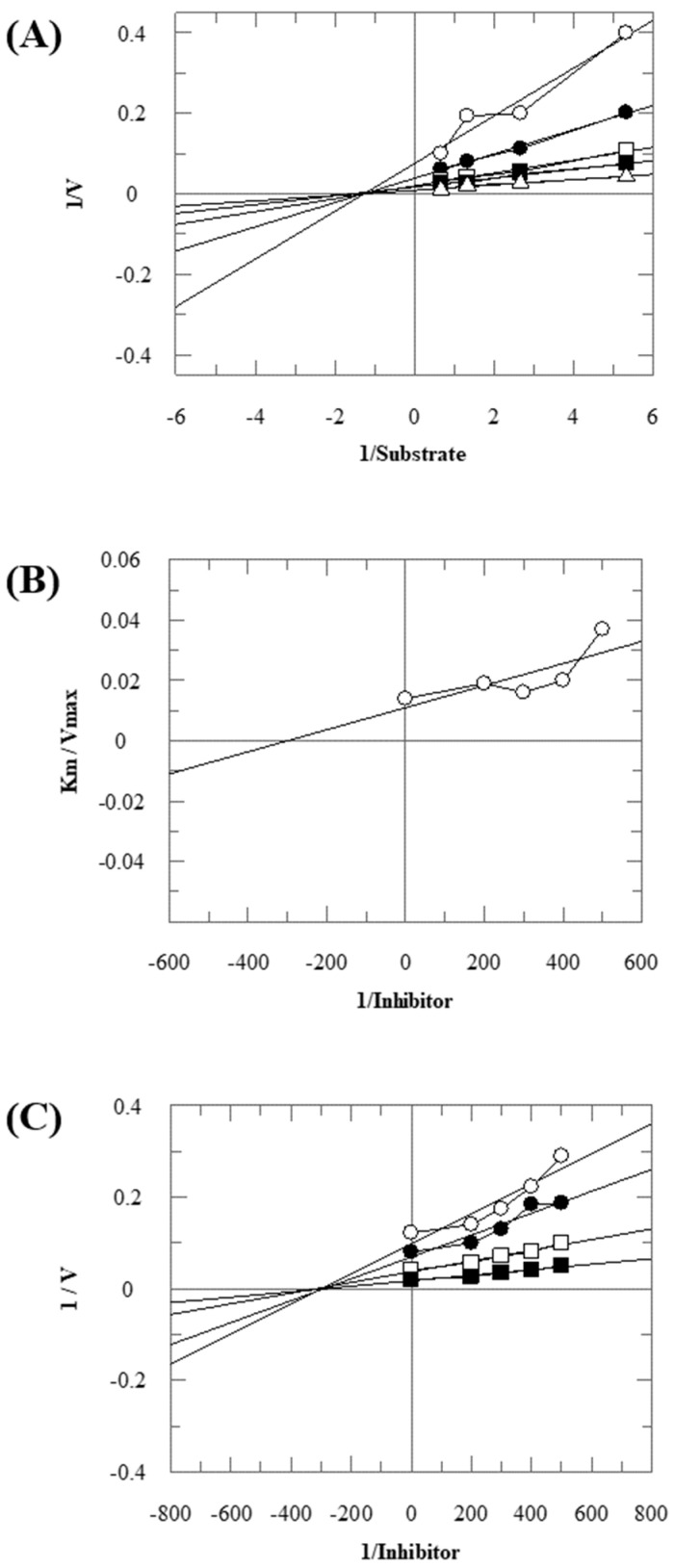
The inhibition of thymidine phosphorylase by compound **12**. (**A**) Lineweaver–Burk plot presented 1/V (velocities) on their Y-axis while that of reciprocal of the substrate (thymidine) on their X-axis. Order of the concentrations of inhibitor included in the absence of compound **12** (∆), in the presence of 200 µM (■), 300 µM (□), 400 µM (●), and 500 µM (○) of compound **12**. (**B**) The secondary replot of Lineweaver–Burk was plotted between the slopes (Km/Vmax) on Y-axis and 1/Inhibitor on X-axis, and (**C**) Dixon plot presented 1/V on Y-axis, while 1/Inhibitor on X-axis.

Compound **12** was found to be the most active analog of this series with IC_50_ = 303.5 ± 0.42 µM. It has a NO_2_ group at the *para* position of the phenyl ring. On the other hand, when the position of NO_2_ was changed from *para* to *meta*, as is the case of compound **38**, it became inactive (showed less than 50% inhibition). This indicates that the *para* position of the NO_2_ group is crucial for TP inhibitory activity. When subjected to mechanistic studies, compound **12** showed a non-competitive type inhibition with Ki = 297.6 ± 0.006 µM (Table 2; Figure 2), suggesting its interaction with amino acid residues other than the active site of the TP enzyme. The dose–response curve of compound **12** with standard deviation is provided in the Supplementary File Figure S1.

**Figure 3:**
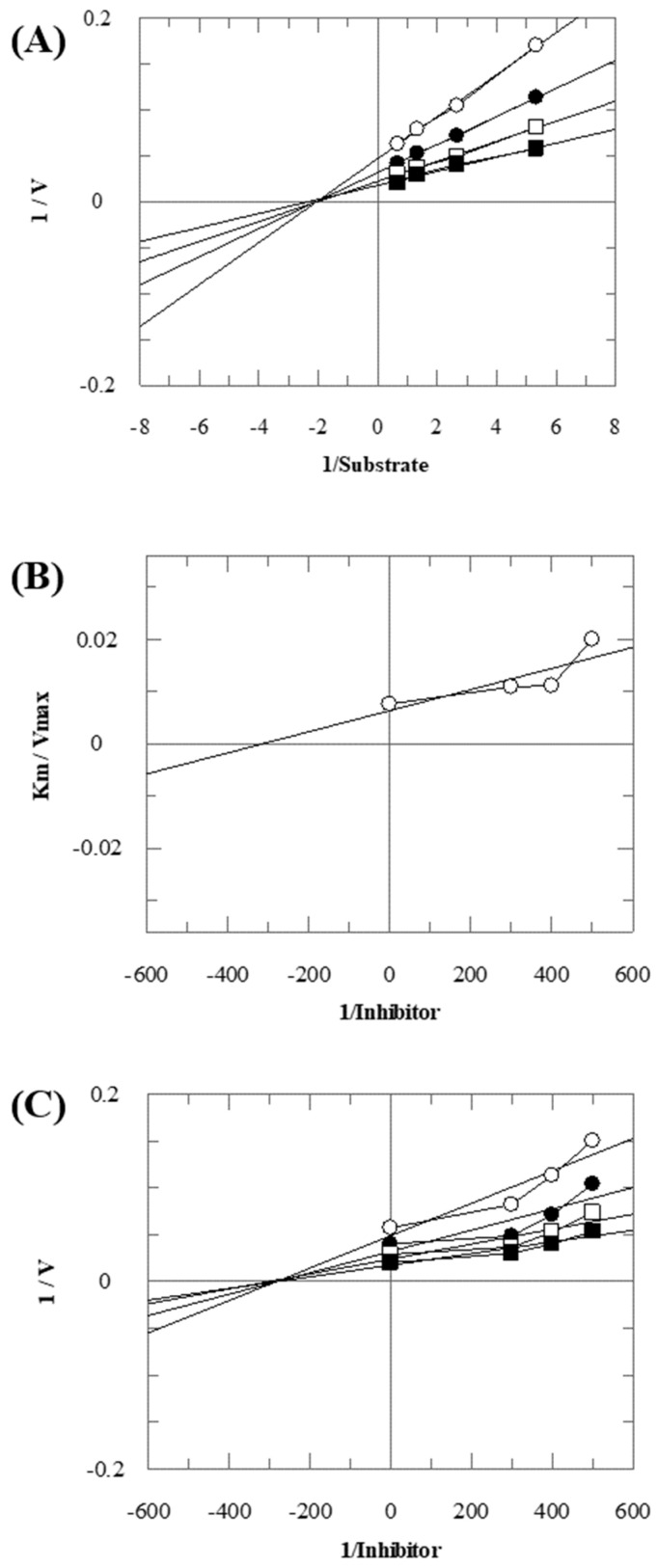
The inhibition of thymidine phosphorylase by compound **33**. (**A**) Lineweaver–Burk plot presented 1/V (velocities) on their Y-axis, while that of reciprocal of the substrate (thymidine) on their X-axis. Order of the concentrations of inhibitor included in the absence of compound **33** (■), in the presence of 300 µM (□), 400 µM (●), and 500 µM (○) of compound **33**. (**B**) The secondary replot of Lineweaver–Burk was plotted between the slopes (Km/Vmax) on Y-axis and 1/Inhibitor on X-axis, and (**C**) Dixon plot presented 1/V on Y-axis, while 1/Inhibitor on X-axis.

Compound **33** was found to be the third most active analog of the series with IC_50_ = 322.6 ± 1.60 µM. It has a trifluoromethyl moiety at the *para* position of the phenyl ring. On the other hand, when a single fluorine atom was substituted at the ortho position of the phenyl ring (i.e., compound **35**), the activity was lost. Mechanistic studies revealed that compound **33** was a non-competitive inhibitor with Ki = 311.4 ± 0.004 µM (Table 2; Figure 3), while the dose–response curve of compound **33** with standard deviation is provided in Supplementary File Figure S1. In compound **34**, the chloro group was substituted at the *para* position of the phenyl ring and showed IC_50_ = 443.9 ± 0.9 µM, less active than compound **33**. *Mono*-*O*-Cl, di-*O*-Cl, and di-*m*-Cl substituted analogs, i.e., compounds **6**, **18**, and **39**, were inactive. Compounds with *para*-bromo substitution (i.e., compound **37**) also showed less than 50% inhibition (i.e., considered as inactive). Interestingly the presence of the bromo group, along with the methoxy group, makes the compound active, as in the case of compound **22** (IC_50_ = 394.3 ± 4.3 µM). Compound **22** has *meta*, *para*-di-chloro, and *meta*-bromo moieties in its skeleton.

Substitution of alkyl groups at the C-4 of the pyrimidine skeleton showed a weak inhibition, as were shown by compounds **10** and **21**. Compound **10** (IC_50_ = 389.0 ± 0.6 µM) possess a butyl group at C-4 of the pyrimidine skeleton, while compound **21** possesses an isobutyl group at C-4 of the pyrimidine skeleton. From these results, it was observed that inhibitory activity increases when the compound possesses an isobutyl group (Compound **21**; IC_50_ = 345.4 ± 0.5 µM). Substitution of the dimethyl amino group at the *para* position of the phenyl ring, as in compound **26**, a weak activity (IC_50_ = 349.9 ± 3.7 µM) was observed.

All active derivatives of the current study were finally subjected to cytotoxicity evaluation. In vitro spectrophotometric assay was employed with a 3T3 (mouse fibroblast) cell line. Results revealed that all active compounds were non-toxic to the cells (Table 1).

### 2.2. In Silico Study

**Figure 4:**
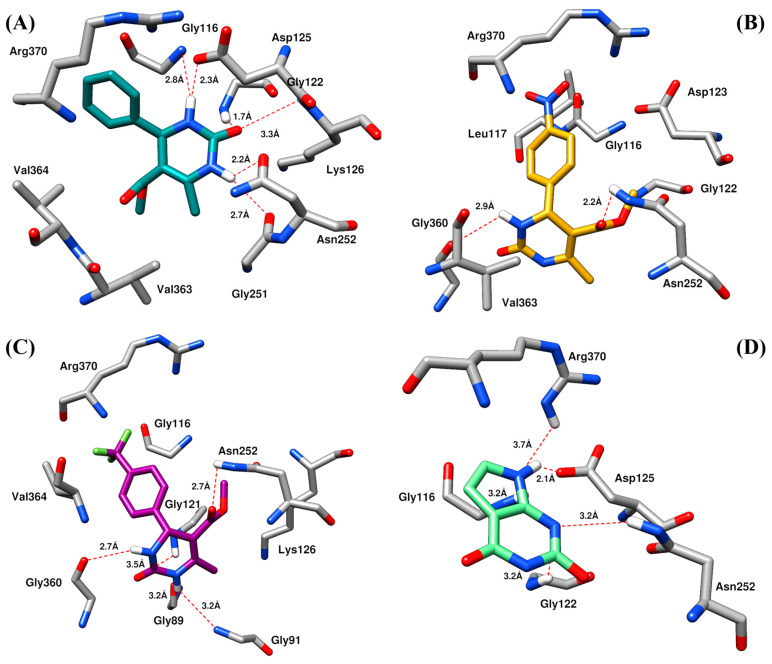
The binding mode of (**A**) compound **1** (**B**) compound **12** (**C**) compound **33,** and (**D**) **7-deazaxanthine** with the allosteric site of TP enzyme predicted by site map analysis. The TP residues are shown in dark grey sticks, while ligands are shown in different color sticks. The red dotted lines represented the hydrogen bond contacts.

Molecular docking is routinely used to predict the binding mode and binding affinity of a small molecule with a receptor protein and to determine the plausible mechanism of observed experimental activities [18,19,20]. Thus, to rationalize the plausible mechanism of observed TP inhibition by dihydropyrimidone derivatives, molecular docking studies were performed using the crystal structure of TP. The details of the selection of the protein target and validation of the docking protocol are provided in the Supplementary File. The binding site was defined on a specific allosteric pocket identified by Bronckaers and co-workers and site map analysis, which is proximal to the catalytic site (Figure S1, Supplementary Materials) [21]. All the selected compounds (**1**, **12**, **33,** and **7-deazaxanthine**) were significantly bound to the allosteric pocket of TP with a binding score in the range of −6.19 kcal/mol to −5.11 kcal/mol (Figure 4, Table 3). The 5′-*O*-tritylinosine (KIN59), a non-competitive inhibitor of human and *E. coli* TP was also docked into the defined binding site. Interestingly, the selected compounds and KIN59 get complexed in the same binding site (Figure S2). As expected, the KIN59 showed the highest binding score of −6.56 kcal/mol in comparison to selected compounds.

Insight into the binding mode of compound **1**, it was observed that the dihydropyrimidone scaffold resides well into the loop region of the binding site, consisting of Gly122, Asp125, Lys126, Asn252, Val363, and Val364 (Figure 4A). Whereas the substituted phenyl ring bends towards the Gly116 and Arg370 by mediating hydrophobic interaction. The carbonyl group and the two nitrogen atoms of the pyrimidine ring were involved in the hydrogen bond contacts with Gly116, Gly122, Asp125, Lys126, Gly251, and Asn252 at a distance of 2.8 Å, 1.7 Å, 2.3 Å, 3.3 Å, 2.7 Å, and 2.2 Å, respectively. Compound **12** exhibited a binding mode comparable to that of compound **1** (Figure 4B). Similarly, a dihydropyrimidone scaffold sandwich between the allosteric loop and an alpha helix consists of Gly122, Asn252, Gly360, and Val363. Similarly, the substituted nitrophenyl ring stacked between the Gly116, Leu117, Asp125, and Arg370 by mediating electrostatic and hydrophobic interactions. In addition, the nitrogen of the pyrimidine ring and ester group substituted on the ring formed hydrogen bonds with Gly360 and Asn252 at a distance of 2.9 Å and 2.2 Å, respectively. In the case of compound **33**, the observed binding mode was also similar to that of compounds **1** and **12** (Figure 4C). The dihydropyrimidone scaffold is stacked between the Gly89, Gly91, Gly121, Lys126, Asn252, and Gly360. Among which Gly89, Gly91, Asn252, and Gly360 were observed to mediate hydrogen bonds with oxygen and nitrogen elements of dihydropyrimidone scaffold at a distance of 3.2 Å, 3.2 Å, 2.7 Å, and 2.7 Å, respectively while rest of the residues were involved in hydrophobic interactions. Whereas the substituted trifluoromethyl moiety at the *para* position of the phenyl ring bends towards the Gly116, Val363, and Asp370 by mediating the hydrophobic interactions. In the case of **7-deazaxanthine**, the pyrimidine ring mediates significant electrostatic and hydrophobic interactions with Gly116, Gly122, Asp125, and Asn252 (Figure 4D). Similarly, the pyrrole ring moves towards the Gly116, Asp125, and Arg370. The nitrogen of pyrimidine and pyrrole ring establishes hydrogen bonds with Gly116, Gly122, Asp125, Asn252, and Arg370 at a distance of 3.2 Å, 3.2 Å, 2.1 Å, 3.2 Å, and 3.7 Å, respectively.

**Figure 5:**
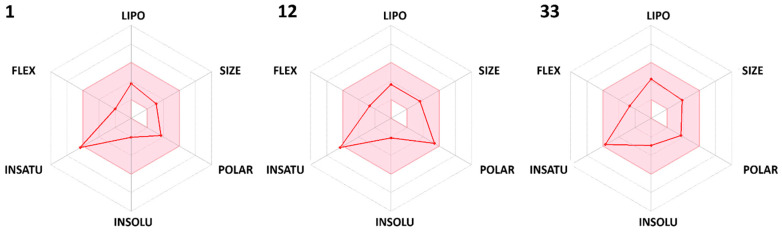
ADME profile of dihydropyrimidone derivatives (**1**, **12,** and **33**) predicted by SwissADME server. The pink region represents the optimal region for each physiochemical property.

Furthermore, the ADME profile of the dihydropyrimidone derivatives (**1**, **12,** and **33**) was evaluated using an online server, SwissADME, to identify the most promising compounds with good pharmacokinetic profiles and the best chances of success in the later phases of drug development [22,23]. Only most potential dihydropyrimidone derivatives have been considered for ADME analysis. Based on six physiochemical properties (solubility, polarity, flexibility, lipophilicity, insaturation, and size) represented in Figure 5, the selected compounds exhibit good oral absorption with good bioavailability scores (0.55) and TPSA (topological polar surface area) values less than 115 Å2, implying that they will be anticipated to be orally absorbed. In terms of pharmacokinetics, all of the selected compounds displayed high gastrointestinal absorption (GI). High GI absorption refers to a prediction that these compounds are likely to be absorbed efficiently from the gastrointestinal tract into the bloodstream after oral administration.

## 3. Material and Methods

Thymidine phosphorylase (source: E. coli, EC Number 2.4.2.4), thymidine (substrate), and potassium phosphate monobasic (as buffer) were acquired from Sigma Aldrich, USA. The **7-deazaxanthine** (standard inhibitor) was acquired from Santa Cruz Biotechnology, Inc., Santa Cruz, CA, USA. Dimethylsulfoxide (DMSO), used as a solvent, was obtained from Fisher Scientific, Schwerte, Germany.

### 3.1. Thymidine Phosphorylase Inhibition Assay

Reaction was performed in 96-well plates and in triplicates. The 200 µL reaction mixture was comprised of 10 µL of the test compound (0.5 mM; dissolved in DMSO), 150 µL of potassium phosphate buffer (pH 7.0, 50 mM), and 20 µL of TP enzyme (0.058 unit/well). The 96-well plate containing all these reagents was then incubated at 30 °C for 10 min. After 10 min, 20 µL of 1.5 mM substrate, i.e., thymidine was added, and changes in O.D. (optical density) were recorded at 290 nm for 10 min by using microtiter plate reader (SpectraMax 384, Molecular Devices, San Jose, CA, USA) [24].

### 3.2. Mechanistic Studies

Kinetic studies are used for the investigation of chemical reactions in terms of their reaction rates, reaction mechanisms, and the factors that affect these parameters [25,26]. Herein, it is used determine the type/mechanism of inhibition of dihydropyrimidone derivatives. In mechanistic studies, TP (0.058 U/200 µL) was incubated with test compounds (200–500 µM) at 30 °C for 10 min. The reaction was then initiated with the addition of different concentrations of thymidine (ranging between 0.1875 and 1.5 mM). Cleavage of thymidine was monitored continuously for 10 min, at 290 nm, by using ELISA plate reader (SpectraMax 384, Molecular Devices, San Jose, CA, USA).

### 3.3. Cytotoxicity

Cytotoxicity of active compounds were evaluated employing a standard MTT calorimetric assay [27]. Mouse fibroblast (3T3 cell line) acquired from American Type Culture Collection (ATCC), Virginia, USA, was cultured in DMEM (Dulbecco’s modified eagle medium). In total, 5% FBS (fetal bovine serum), penicillin (100 IU/mL), and streptomycin (100 mg/mL) were then added to the medium in 75 cm^3^ flasks, and kept in a CO_2_ incubator at 37 °C. The harvested cell was then diluted with a specific medium. Haemocytometer was used for the quantification of cells. After that, the culture of cell having concentration of 5x104 (cells/mL) was prepared, inoculated into 96-well plates (100 µL/well), and then incubated overnight. After incubation, the old medium was removed, and fresh medium (200 µL) was added. Then different concentrations of test compounds (ranging from 1 to 30 µM) were added. After incubation of 48 h, each well received 200 µL of MTT dye (0.5 mg/mL), and further incubated for a duration of 4 h. After 4 h, 100 µL of DMSO was introduced into each well of the 96-well plate. The reduction of MTT to formazan was measured by recording absorbance at 540 nm with the help of a microplate reader (SpectraMax 384, Molecular Devices, San Jose, CA, USA).

### 3.4. Statistical Analysis

SoftMax Pro 4.8 software (Molecular Devices, San Jose, CA, USA) was used to analyse the results. Percent inhibition was calculated by using Microsoft Excel by applying the following formula:

% Inhibition=100−(O.D.Test compound/O.D.Control)×100

where O.D. represented the optical density.

Results were presented as means ± SEM (standard error of mean) (n = 3). EZ-FIT, enzyme kinetics software (Perrella Scientific, Inc., Amherst, GA, USA) was used to calculate the IC_50_ values. In order to analyse the kinetic parameter, Grafit version 7.0 (Erithacus Software Ltd., Horley, UK) was used.

### 3.5. Molecular Docking Studies

Molecular docking study was conducted to investigate the potential mechanism of TP inhibition. The crystal structure of Thymidine Phosphorylase in complex with an inhibitor, 3′-azido-2′-fluoro-2′,3′-dideoxyuridine reported by Timofeev and co-workers was used as a target protein (PDB ID 4EAD) [28]. The compounds of interest were sketched in MOE v.2019 using the Builder module and then subjected to structure correction, protonation, and energy minimization using the MMFF94x force field. The crystal structure of the target protein was retrieved from the RCSB Protein Data Bank and prepared for docking by placing hydrogens and assigning ionization states and then minimizing the structure using Amber99 force field. The results of kinetic studies showed that the most potential inhibitors displayed a non-competitive mode of inhibition. Therefore, to investigate potential allosteric sites for these inhibitors, site map analysis was conducted using the Site Finder module of MOE. Site map analysis involves the use of computational methods to predict potential binding sites on a protein based on its structure and electrostatic properties. The site map analysis identified 26 potential allosteric sites on thymidine phosphorylase and the site with the highest PLB (propensity for ligand binding) score was selected for further analysis. Finally, the ligands were docked into the selected allosteric site to investigate their binding interactions and potential as inhibitors of thymidine phosphorylase. Previously utilized protocol by Uddin and co-workers for the docking studies of inhibitors of TP was utilized [29]. Therefore, Triangular Matcher was used as a placement method with London dG and GBVI/WSA dG as scoring and rescoring functions, respectively. For each compound, ten conformations were generated and five best were retained for analysis. All the graphics were rendered using Chimera software (UCSF, San Francisco, CA, USA) [30].

### 3.6. ADME Analysis

SwissADME (Swiss Institute of Bioinformatics (SIB), Geneva, Switzerland) is an online server that predicts several ADME (absorption, distribution, metabolism, and excretion) properties of small molecules [31]. The server provides estimates of physicochemical properties, lipophilicity, drug-likeness, and pharmacokinetic properties of small molecules. Herein, the SwissADME server was utilized to predict the ADME profile of potential dihydropyrimidone derivatives (compound **1**, compound **12**, and compound **33**). In this regard, the canonical SMILES of selected compounds were subjected to the server.

## 4. Conclusions

Present study has identified dihydropyrimidone derivatives as potential inhibitors of thymidine phosphorylase enzyme. Among different derivatives, compounds **1**, **12**, and **33** showed inhibition of thymidine phosphorylase with non-competitive mode. Additionally, these compounds were found to be non-toxic to 3T3 (mouse fibroblast) cell line. Furthermore, molecular docking studies inferred that the dihydropyrimidone derivatives significantly reside well into the binding site of TP, which is proximal to catalytic site. In addition, ADME analysis of dihydropyrimidone derivatives suggested the good oral bioavailability and high gastrointestinal absorption. However, these initial hits need to be structurally optimized to improve their potency and selectivity for thymidine phosphorylase inhibition. Once optimized, these compounds could potentially be studied for their effect on angiogenesis, which is an important process in tumor growth and metastasis. Overall, this study provides a promising starting point for the development of new inhibitors of thymidine phosphorylase and highlights the potential of dihydropyrimidone derivatives as a class of compounds with therapeutic activity.

## Figures and Tables

**Table 1:** Thymidine phosphorylase (TP) inhibitory activity of dihydropyrimidone (**1**–**40**).

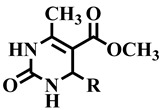					
Compound Number	R	Thymidine Phosphorylase Inhibitory Activity		Cytotoxicity Studies	
		% Inhibition	IC_50_ ± SEM (µM)	Cell Viability (%)	IC_50_ ± SD *(*µM)
**1**	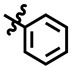 Methyl 6-methyl-2-oxo-4-phenyl-1,2,3,4-tetrahydropyrimidine-5-carboxylate	84.0	314.3 ± 0.9	57	NC
**2**	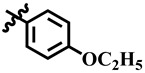 Methyl 4-(4-ethoxyphenyl)-6-methyl-2-oxo-1,2,3,4-tetrahydropyrimidine-5-carboxylate	78.2	389.2 ± 6.2	67	NC
**3**	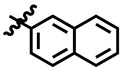 Methyl 6-methyl-4-(naphthalen-2-yl)-2-oxo- 1,2,3,4-tetrahydropyrimidine-5-carboxylate	38.1	N/A	NC	NC
**4**	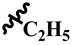 Methyl 4-ethyl-6-methyl-2-oxo-1,2,3,4- tetrahydropyrimidine-5-carboxylate	46.8	N/A	NC	NC
**5**	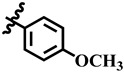 Methyl 4-(4-methoxyphenyl)-6-methyl-2-oxo-1,2,3,4-tetrahydropyrimidine-5-carboxylate	52.9	387.4 ± 2.0	72	NC
**6**	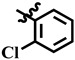 Methyl 4-(2-chlorophenyl)-6-methyl-2-oxo-1,2,3,4-tetrahydropyrimidine-5-carboxylate	−65.3	N/A	NC	NC
**7**	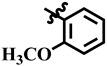 Methyl 4-(2-methoxyphenyl)-6-methyl-2-oxo-1,2,3,4-tetrahydropyrimidine-5-carboxylate	−19.3	N/A	NC	NC
**8**	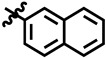 Methyl 6-methyl-4-(naphthalen-2-yl)-2-oxo-1,2,3,4-tetrahydropyrimidine-5-carboxylate	−10.3	N/A	NC	NC
**9**	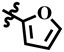 Methyl 4-(furan-2-yl)-6-methyl-2-oxo-1,2,3,4-tetrahydropyrimidine-5-carboxylate	78.9	373.6 ± 2.2	72	NC
**10**	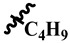 Methyl 4-butyl-6-methyl-2-oxo-1,2,3,4-tetrahydropyrimidine-5-carboxylate	92.7	389.0 ± 0.6	66	NC
**11**	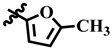 Methyl 6-methyl-4-(5-methylfuran-2-yl)-2-oxo-1,2,3,4-tetrahydropyrimidine-5-carboxylate	76.9	386.0 ± 0.6	77	NC
**12**	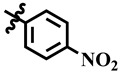 Methyl 6-methyl-4-(4-nitrophenyl)-2-oxo-1,2,3,4-tetrahydropyrimidine-5-carboxylate	91.5	303.5 ± 0.4	57	NC
**13**	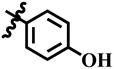 Methyl 4-(4-hydroxyphenyl)-6-methyl-2-oxo-1,2,3,4-tetrahydropyrimidine-5-carboxylate	56.2	448.9 ± 4.1	72	NC
**14**	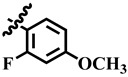 Methyl 4-(2-fluoro-4-methoxyphenyl)-6-methyl-2-oxo-1,2,3,4-tetrahydropyrimidine-5-carboxylate	68.4	397.1 ± 0.1	71	NC
**15**	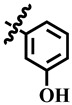 Methyl 4-(3-hydroxyphenyl)-6-methyl-2-oxo-1,2,3,4-tetrahydropyrimidine-5-carboxylate	38.9	NA	NC	NC
**16**	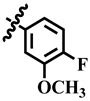 Methyl 4-(4-fluoro-3-methoxyphenyl)-6-methyl-2-oxo-1,2,3,4-tetrahydropyrimidine-5-carboxylate	47.8	NA	NC	NC
**17**	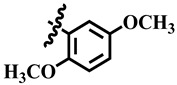 Methyl 4-(2,5-dimethoxyphenyl)-6-methyl-2-oxo-1,2,3,4-tetrahydropyrimidine-5-carboxylate	46.5	NA	NC	NC
**18**	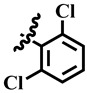 Methyl 4-(2,6-dichlorophenyl)-6-methyl-2-oxo-1,2,3,4-tetrahydropyrimidine-5-carboxylate	−15.4	NA	NC	NC
**19**	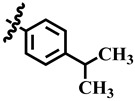 Methyl 4-(4-isopropylphenyl)-6-methyl-2-oxo-1,2,3,4-tetrahydropyrimidine-5-carboxylate	−18.5	NA	NC	NC
**20**	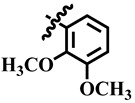 Methyl 4-(2,3-dimethoxyphenyl)-6-methyl-2-oxo-1,2,3,4-tetrahydropyrimidine-5-carboxylate	67.8	414.7 ± 1.6	62	NC
**21**	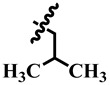 Methyl 4-isobutyl-6-methyl-2-oxo-1,2,3,4-tetrahydropyrimidine-5-carboxylate	90.7	345.4 ± 0.5	55	NC
**22**	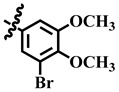 Methyl 4-(3-bromo-4,5-dimethoxyphenyl)-6-methyl-2-oxo-1,2,3,4-tetrahydropyrimidine-5-carboxylate	61.9	394.3 ± 4.3	76	NC
**23**	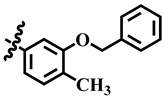 Methyl 4-(3-(benzyloxy)-4-methoxyphenyl)-6-methyl-2-oxo-1,2,3,4-tetrahydropyrimidine-5-carboxylate	7.5	NA	NC	NC
**24**	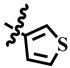 Methyl 6-methyl-2-oxo-4-(thiophen-3-yl)-1,2,3,4-tetrahydropyrimidine-5-carboxylate	79.6	350.6 ± 0.6	82	NC
**25**	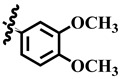 Methyl 4-(3,4-dimethoxyphenyl)-6-methyl-2-oxo-1,2,3,4-tetrahydropyrimidine-5-carboxylate	59.0	424.1 ± 0.9	99	NC
**26**	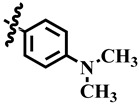 Methyl 4-(4-(dimethylamino) phenyl)-6-methyl-2-oxo-1,2,3,4-tetrahydropyrimidine-5-carboxylate	92.3	349.9 ± 3.7	57	NC
**27**	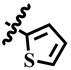 Methyl 6-methyl-2-oxo-4-(thiophen-2-yl)-1,2,3,4-tetrahydropyrimidine-5-carboxylate	47.1	NA	NC	NC
**28**	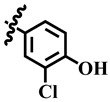 Methyl 4-(3-chloro-4-hydroxyphenyl)-6-methyl-2-oxo-1,2,3,4-tetrahydropyrimidine-5-carboxylate	67.5	404.6 ± 1.0	95	NC
**29**	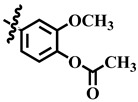 Methyl 4-(4-acetoxy-3-methoxyphenyl)-6-methyl-2-oxo-1,2,3,4-tetrahydropyrimidine-5-carboxylate	76.0	400.5 ± 0.6	80	NC
**30**	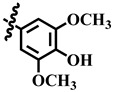 Methyl 4-(4-hydroxy-3,5-dimethoxyphenyl)-6-methyl-2-oxo-1,2,3,4-tetrahydropyrimidine-5-carboxylate	69.6	396.7 ± 1.5	75	NC
**31**	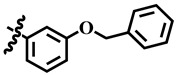 Methyl 4-(3-(benzyloxy) phenyl)-6-methyl-2-oxo-1,2,3,4-tetrahydropyrimidine-5-carboxylate	−41.9	NA	NC	NC
**32**	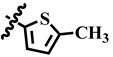 Methyl 6-methyl-4-(5-methylthiophen-2-yl)-2-oxo-1,2,3,4-tetrahydropyrimidine-5-carboxylate	58.3	485.7 ± 1.5	85	NC
**33**	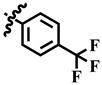 Methyl 6-methyl-2-oxo-4-(4-(trifluoromethyl) phenyl)-1,2,3,4-tetrahydropyrimidine-5-carboxylate	73.6	322.6 ± 1.6	61	NC
**34**	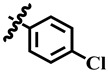 Methyl 4-(4-chlorophenyl)-6-methyl-2-oxo-1,2,3,4-tetrahydropyrimidine-5-carboxylate	66.3	443.9 ± 0.9	72	NC
**35**	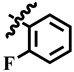 Methyl 4-(2-fluorophenyl)-6-methyl-2-oxo-1,2,3,4-tetrahydropyrimidine-5-carboxylate	9.6	NA	NC	NC
**36**	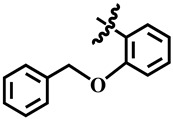 Methyl 4-(2-(benzyloxy) phenyl)-6-methyl-2-oxo-1,2,3,4-tetrahydropyrimidine-5-carboxylate	5.3	NA	NC	NC
**37**	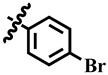 Methyl 4-(4-bromophenyl)-6-methyl-2-oxo-1,2,3,4-tetrahydropyrimidine-5-carboxylate	15.7	NA	NC	NC
**38**	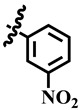 Methyl 6-methyl-4-(3-nitrophenyl)-2-oxo-1,2,3,4-tetrahydropyrimidine-5-carboxylate	−23.1	NA	NC	NC
**39**	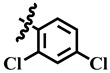 Methyl 4-(2,4-dichlorophenyl)-6-methyl-2-oxo-1,2,3,4-tetrahydropyrimidine-5-carboxylate	−1.8	NA	NC	NC
**40**	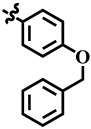 Methyl 4-(4-(benzyloxy) phenyl)-6-methyl-2-oxo-1,2,3,4-tetrahydropyrimidine-5-carboxylate	−273.6	NA	NC	NC
Standard*	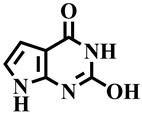 7-Deazaxanthine	82.0	41.0 ± 1.6	NC	NC
Standard*	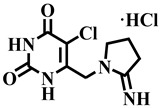 Tipiracil-HCl	92.0	0.014 ± 0.04	NC	NC
Standard**	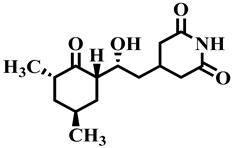 Cycloheximide	ND	ND	30	0.8 ± 0.2

SEM: standard error of the mean (n = 3); SD: standard deviation; NA: not active; NC: non-cytotoxic; ND: not determine; Standard*: used as reference compound for in vitro assay; Standard**: used as standard in cytotoxicity assay.

**Table 2:** Kinetics parameters of selected potent compounds.

Compound	*Ki* ± SEM (µM)	Type of Inhibition
**1**	326.3 ± 0.002	Non-Competitive
**12**	297.6 ± 0.006	Non-Competitive
**33**	311.4 ± 0.004	Non-Competitive
**7-deazaxanthine**	45.0 ± 0.003	Non-Competitive

**7-deazaxanthine**: standard; SEM: standard error of the mean; *Ki*: dissociation constant.

**Table 3:** Detail of results of molecular docking studies including docking scores, type of interactions, and interacted residues.

Compound	Docking Score (kcal/mol)	Type of Interactions	
		Hydrophobic Interactions	Hydrogen Bonding
**1**	−6.19	Gly116, Gly122, Asp125, Lys126, Asn252, Arg370, Val363, and Val364	Gly116, Gly122, Asp125, Lys126, and Asn252
**12**	−5.60	Gly116, Leu117, Gly122, Asp125, Asn252, Gly360, Arg370, and Val363	Asn252 and Gly360
**33**	−5.11	Gly116, Gly121, Lys126, Asn252, Val363, and Asp370	Gly89, Gly91, Asn252, and Gly360
**7-deazaxanthine**	−5.23	Gly116 and Gly122	Gly116, Gly122, Asp125, Asn252, and Arg370

## Data Availability

Not applicable.

## Supplementary Materials

The following supporting information can be downloaded at: https://www.mdpi.com/article/10.3390/molecules28083634/s1, Figure S1: Dose response curve of compounds with standard deviation; Figure S2: Comparison of allosteric sites and binding modes of compounds on human and E. coli Thymidine Phosphorylase. References [32,33,34] are cited in the Supplementary Materials.

## Sample Availability

Samples of the compounds are not available from the authors.

## References

[1] Panigrahy D., Singer S., Shen L.Q., Butterfield C.E., Freedman D.A., Chen E.J., Moses M.A., Kilroy S., Duensing S., Fletcher C. (2002). PPARγ Ligands Inhibit Primary Tumor Growth and Metastasis by Inhibiting Angiogenesis. J. Clin. Invest..

[2] Vives M., Ginestà M.M., Gracova K., Graupera M., Casanovas O., Capellà G., Serrano T., Laquente B., Viñals F. (2013). Metronomic Chemotherapy Following the Maximum Tolerated Dose Is an Effective Anti-Tumour Therapy Affecting Angiogenesis, Tumour Dissemination and Cancer Stem Cells. Int. J. Cancer.

[3] Schwartz P.A., Vetticatt M.J., Schramm V.L. (2011). Transition State Analysis of the Arsenolytic Depyrimidination of Thymidine by Human Thymidine Phosphorylase. Biochemistry.

[4] Matsushita S., Nitanda T., Furukawa T., Sumizawa T., Tani A., Nishimoto K., Akiba S., Miyadera K., Fukushima M., Yamada Y. (1999). The Effect of a Thymidine Phosphorylase Inhibitor on Angiogenesis and Apoptosis in Tumors. Cancer Res..

[5] Bronckaers A., Gago F., Balzarini J., Liekens S. (2009). The Dual Role of Thymidine Phosphorylase in Cancer Development and Chemotherapy. Med. Res. Rev..

[6] Mitsiki E., Papageorgiou A.C., Iyer S., Thiyagarajan N., Prior S.H., Sleep D., Finnis C., Acharya K.R. (2009). Structures of Native Human Thymidine Phosphorylase and in Complex with 5-Iodouracil. Biochem. Biophys. Res. Commun..

[7] Focher F., Spadari S. (2001). Thymidine Phosphorylase: A Two-Face Janus in Anticancer Chemotherapy. Curr. Cancer Drug. Targets.

[8] Almasmoum H. (2021). Characterization of Mucin 2 Expression in Colorectal Cancer with and without Chemotherapies, in Vivo and in Vitro Study. JUQUMS.

[9] Mayer R.J., Van Cutsem E., Falcone A., Yoshino T., Garcia-Carbonero R., Mizunuma N., Yamazaki K., Shimada Y., Tabernero J., Komatsu Y. (2015). Randomized Trial of TAS-102 for Refractory Metastatic Colorectal Cancer. N. Engl. J. Med..

[10] Yano S., Kazuno H., Sato T., Suzuki N., Emura T., Wierzba K., Yamashita J., Tada Y., Yamada Y., Fukushima M. (2004). Synthesis and Evaluation of 6-Methylene-Bridged Uracil Derivatives. Part 2: Optimization of Inhibitors of Human Thymidine Phosphorylase and Their Selectivity with Uridine Phosphorylase. Bioorg Med. Chem..

[11] Balzarini J., Gamboa A.E., Esnouf R., Liekens S., Neyts J., De Clercq E., Camarasa M.J., Pérez-Pérez M.J. (1998). 7-Deazaxanthine, a Novel Prototype Inhibitor of Thymidine Phosphorylase. FEBS Lett..

[12] Sun L., Li J., Bera H., Dolzhenko A.V., Chiu G.N.C., Chui W.K. (2013). Fragment-Based Approach to the Design of 5-Chlorouracil-Linked-Pyrazolo [1,5-a][1,3,5]Triazines as Thymidine Phosphorylase Inhibitors. Eur. J. Med. Chem..

[13] Bera H., Tan B.J., Sun L., Dolzhenko A.V., Chui W.-K., Chiu G.N.C. (2013). A Structure-Activity Relationship Study of 1,2,4-Triazolo [1,5-a][1,3,5]Triazin-5,7-Dione and Its 5-Thioxo Analogues on Anti-Thymidine Phosphorylase and Associated Anti-Angiogenic Activities. Eur. J. Med. Chem..

[14] Bera H., Chui W.-K., Gupta S.D., Dolzhenko A.V., Sun L. (2013). Synthesis, in Vitro Evaluation of Thymidine Phosphorylase Inhibitory Activity, and in Silico Study of 1,3,5-Triazin-2,4-Dione and Its Fused Analogues. Med. Chem. Res..

[15] Dorababu A. (2019). Evolution of Uracil Based Thymidine Phosphorylase Inhibitors, SAR and Electronic Correlation: Revisit. Drug. Dev. Res..

[16] Ali F., Khan K.M., Salar U., Iqbal S., Taha M., Ismail N.H., Perveen S., Wadood A., Ghufran M., Ali B. (2016). Dihydropyrimidones: As Novel Class of β-Glucuronidase Inhibitors. Bioorganic Med. Chem..

[17] Dimas K., Demetzos C., Marsellos M., Sotiriadou R., Malamas M., Kokkinopoulos D. (1998). Cytotoxic Activity of Labdane Type Diterpenes against Human Leukemic Cell Lines in Vitro. Planta Med..

[18] Abd Alhameed R., Almarhoon Z., Sholkamy E.N., Ali Khan S., Ul-Haq Z., Sharma A., de la Torre B.G., Albericio F., El-Faham A. (2020). Novel 4,6-Disubstituted s-Triazin-2-Yl Amino Acid Derivatives as Promising Antifungal Agents. J. Fungi.

[19] Aldarhami A., Bazaid A.S., Alhamed A.S., Alghaith A.F., Ahamad S.R., Alassmrry Y.A., Alharazi T., Snoussi M., Qanash H., Alamri A. (2023). Antimicrobial Potential of *Pithecellobium dulce* Seed Extract against Pathogenic Bacteria: In Silico and In Vitro Evaluation. BioMed. Res. Int..

[20] Alfaifi G.H., Farghaly T.A., Abdellattif M.H. (2023). Indenyl-Thiazole and Indenyl-Formazan Derivatives: Synthesis, Anticancer Screening Studies, Molecular-Docking, and Pharmacokinetic/ Molin-Spiration Properties. PLoS ONE.

[21] Bronckaers A., Aguado L., Negri A., Camarasa M.-J., Balzarini J., Pérez-Pérez M.-J., Gago F., Liekens S. (2009). Identification of Aspartic Acid-203 in Human Thymidine Phosphorylase as an Important Residue for Both Catalysis and Non-Competitive Inhibition by the Small Molecule “Crystallization Chaperone” 5′-O-Tritylinosine (KIN59). Biochem. Pharmacol..

[22] Hosea N.A., Jones H.M. (2013). Predicting Pharmacokinetic Profiles Using in Silico Derived Parameters. Mol. Pharm..

[23] Bakhdar F. (2020). The Role of CYP 450 Isozymes in Drug-Drug Interaction. JUQUMS.

[24] Bera H., Dolzhenko A.V., Sun L., Dutta Gupta S., Chui W.-K. (2013). Synthesis and in Vitro Evaluation of 1,2,4-Triazolo [1,5-a][1,3,5]Triazine Derivatives as Thymidine Phosphorylase Inhibitors. Chem. Biol. Drug. Des..

[25] Iqubal S.M.S. (2022). Review on Kinetic Studies of α-Hydroxy Acids (Glycolic, Mandelic, Citric, Tartaric and Malic) and Some Other Organic Compounds with Water Soluble Nano Particles of Colloidal MnO2 in Absence and Presence of Non-Ionic Surfactant (TX-100). J. Umm. Al-Qura. Univ. Appll. Sci..

[26] Whiteley C.G. (2000). Mechanistic and Kinetic Studies of Inhibition of Enzymes. Cell Biochem. Biophys..

[27] Vega-Avila E., Pugsley M.K. (2011). An Overview of Colorimetric Assay Methods Used to Assess Survival or Proliferation of Mammalian Cells. Proc. West. Pharmacol. Soc..

[28] Timofeev V.I., Abramchik Y.A., Fateev I.V., Zhukhlistova N.E., Murav’eva T.I., Kuranova I.P., Esipov R.S. (2013). Three-Dimensional Structure of Thymidine Phosphorylase from E. Coli in Complex with 3′-Azido-2′-Fluoro-2′,3′-Dideoxyuridine. Crystallogr. Rep..

[29] Uddin I., Taha M., Rahim F., Wadood A. (2018). Synthesis and Molecular Docking Study of Piperazine Derivatives as Potent Inhibitor of Thymidine Phosphorylase. Bioorganic Chem..

[30] Goddard T.D., Huang C.C., Ferrin T.E. (2005). Software Extensions to UCSF Chimera for Interactive Visualization of Large Molecular Assemblies. Structure.

[31] Daina A., Michielin O., Zoete V. (2017). SwissADME: A Free Web Tool to Evaluate Pharmacokinetics, Drug-Likeness and Medicinal Chemistry Friendliness of Small Molecules. Sci. Rep..

[32] Shahzad S.A., Yar M., Bajda M., Jadoon B., Khan Z.A., Naqvi S.A.R., Shaikh A.J., Hayat K., Mahmmod A., Mahmood N. (2014). Synthesis and Biological Evaluation of Novel Oxadiazole Derivatives: A New Class of Thymidine Phosphorylase Inhibitors as Potential Anti-Tumor Agents. Bioorg. Med. Chem..

[33] Almandil N.B., Taha M., Farooq R.K., Alhibshi A., Ibrahim M., Anouar E.H., Gollapalli M., Rahim F., Nawaz M., Shah S.A.A. (2019). Synthesis of Thymidine Phosphorylase Inhibitor Based on Quinoxaline Derivatives and Their Molecular Docking Study. Molecules.

[34] Liekens S., Hernández A.-I., Ribatti D., De Clercq E., Camarasa M.-J., Pérez-Pérez M.-J., Balzarini J. (2004). The Nucleoside Derivative 5′-*O*-Trityl-Inosine (KIN59) Suppresses Thymidine Phosphorylase-Triggered Angiogenesis via a Noncompetitive Mechanism of Action*. J. Biol. Chem..

