# Supplementary material for: Dihydropyrimidone Derivatives as Thymidine Phosphorylase Inhibitors: Inhibition Kinetics, Cytotoxicity, and Molecular Docking
Source: Molecules. 2023 Apr 21;28(8):3634. doi: 10.3390/molecules28083634 (PMC10143232; doi:10.3390/molecules28083634)
Supplement: Supplementary file 1 [file molecules-28-03634-s001.zip › molecules-2294357-supplementary.pdf]

Supplementary Information

Dihydropyrimidone Derivatives as Thymidine Phosphorylase Inhibitors: Inhibition Kinetics, Cytotoxicity, and Molecular Docking

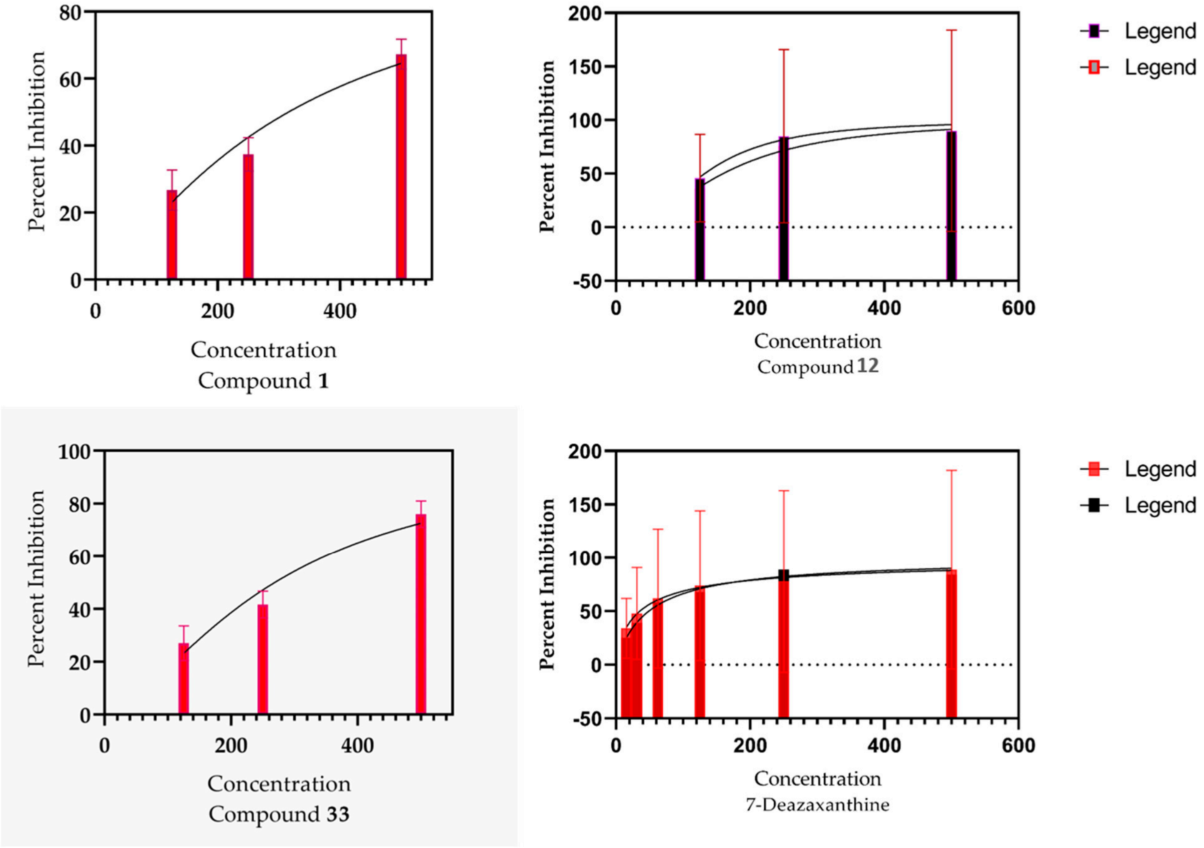

Figure S1. Dose–response curve of compound **1**, **12**, **33,** and **7-deazaxanthine** with standard deviation.

Molecular Docking Target and Protocol Validation

Molecular docking is a computational technique used to predict the binding mode and binding affinity of a small molecule (ligand) with a receptor protein. Molecular docking has been extensively used to explore the inhibition mechanism of Thymidine Phosphorylase (TP) inhibitors, by predicting the binding modes and binding affinities of small molecules to the active site of TP. In 2014, Shahzad and co-workers synthesized oxadiazole derivatives and evaluate their thymidine phosphorylase/PD-ECGF (*E. coli*) activity by measuring the absorbance at 290 nm spectrophotometrically. Subsequently,

the docking studies were carried out to determine the plausible mechanism of TP inhibition by using the crystal structure of TP of *E. coli* (PDB ID 4EAD) [1]. Similarly, in 2018, Uddin and co-workers synthesized piperazine derivatives and evaluate their thymidine phosphorylase/PD-ECGF (*E. coli*) activity by measuring the absorbance at 290 nm spectrophotometrically. Afterward, they determine the inhibition mechanism by molecular docking studies using the crystal structure of TP of *E. coli* (PDB ID 4EAD) [2]. A similar protocol was also utilized by Almandil and co-workers to rationalize the TP inhibitory potential of quinoxaline derivatives [3]. Herein, we also utilized the TP from *E. coli* to determine the TP inhibitory potential of previously synthesized dihydropyrimidone derivatives. Therefore, we also utilized the crystal structure of TP of *E. coli* (PDB ID 4EAD) to evaluate the binding mechanism of dihydropyrimidone derivatives.

Since the most potent inhibitors showed the non- and uncompetitive mode of inhibition in kinetic studies, we searched for the allosteric binding site of TP. However, we did not find any allosteric inhibition mechanism or allosteric binding site of TP of *E. coli* in the literature. However, Bronckaers and co-workers investigated the non-competitive human TP inhibition mechanism of 5′-O-tritylinosine (KIN59), an inhibitor of TP [4]. The TP-catalyzed conversion of dThd to Thy was inhibited by KIN59 at a 50% inhibitory concentration (IC_{50}) of 44 ± 3 and 67 ± 20 μM, respectively, when purified *E. coli* and human TP were used as the enzyme source. KIN59 does not compete with the pyrimidine nucleoside or the phosphate-binding site of the enzyme but noncompetitively inhibits TP when thymidine or phosphate is used as the variable substrate [5]. According to the Bronckaers and co-workers' study, a cavity was generated in the vicinity of the Gly405–Val419 (correspond to Gly367-Val381 of *E. coli* TP) loop that was mainly lined by the side chains of apolar residues Leu-155 (Leu124 of *E. coli* TP), Val166 (Ile135 of *E. coli* TP) and Ile167 (Phe136 of *E. coli* TP), and positively charged residues Arg146 (Arg115 of *E. coli* TP) and Arg408 (Arg370 of *E. coli* TP), all of which can provide good anchoring points for the crucial trityl group of KIN59. In the general primary sequence of TP is mostly conserved throughout the evolution and mammalian TP share 39% sequence similarity with TP of *E. coli*. The mammalian enzyme also shares 65–70% similarity with the 3D structure (Figure S2A) and allosteric site residues (Figure S2B), identified by Bronckaers and co-workers of *E. coli* TP enzyme. Therefore, we docked the KIN59 to validate the docking protocol. For the selection of the binding site, site map analysis was performed. Interestingly, the site map analysis identified the allosteric site, which is proximal to its catalytic site having the highest PLB (propensity for ligand binding) score and consists of all the crucial residues that were previously identified by Bronckaers and co-workers.

The KIN59 significantly interacts with the Gly116, Leu117, Gly122, Asp172, Arg370, Gly367, and Val381 with a binding affinity of -6.56 kcal/mol (Figure S2C). The same site was selected for the docking studies of dihydropyrimidone derivatives. It was interesting to observe that compounds **1**, **12,** and **33** also occupied the same site such as KIN59 (Figure S2C).

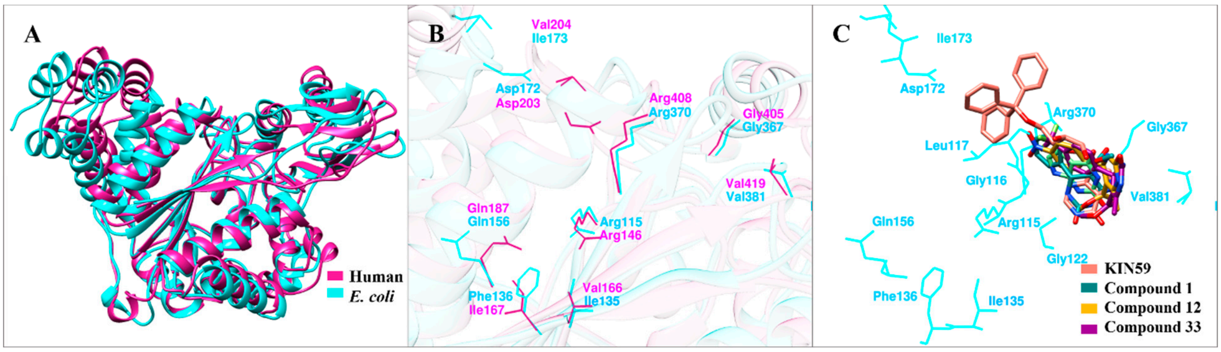

Figure S2. (**A**) Alignment of human TP (PDB ID 2J0F) and *E. coli* TP (PDB ID 4EAD). (**B**) Close view of alignment of allosteric site of human and *E. coli* TP. (**C**) Binding mode of compounds **1**, **12**, and **33** superimposed on the KIN59 complex.

References

1. Shahzad, S.A.; Yar, M.; Bajda, M.; Jadoon, B.; Khan, Z.A.; Naqvi, S.A.R.; Shaikh, A.J.; Hayat, K.; Mahmmod, A.; Mahmood, N.; et al. Synthesis and Biological Evaluation of Novel Oxadiazole Derivatives: A New Class of Thymidine Phosphorylase Inhibitors as Potential Anti-Tumor Agents. *Bioorganic & Medicinal Chemistry* **2014**, *22*, 1008–1015, doi:10.1016/j.bmc.2013.12.043.
2. Uddin, I.; Taha, M.; Rahim, F.; Wadood, A. Synthesis and Molecular Docking Study of Piperazine Derivatives as Potent Inhibitor of Thymidine Phosphorylase. *Bioorganic Chemistry* **2018**, *78*, 324–331, doi:10.1016/j.bioorg.2018.03.026.
3. Almandil, N.B.; Taha, M.; Farooq, R.K.; Alhibshi, A.; Ibrahim, M.; Anouar, E.H.; Gollapalli, M.; Rahim, F.; Nawaz, M.; Shah, S.A.A.; et al. Synthesis of Thymidine Phosphorylase Inhibitor Based on Quinoxaline Derivatives and Their Molecular Docking Study. *Molecules* **2019**, *24*, 1002, doi:10.3390/molecules24061002.
4. Bronckaers, A.; Aguado, L.; Negri, A.; Camarasa, M.-J.; Balzarini, J.; Pérez-Pérez, M.-J.; Gago, F.; Liekens, S. Identification of Aspartic Acid-203 in Human Thymidine Phosphorylase as an Important Residue for Both Catalysis and Non-Competitive Inhibition by the Small Molecule “Crystallization Chaperone” 5′-O-Tritylinosine (KIN59). *Biochemical Pharmacology* **2009**, *78*, 231–240, doi:10.1016/j.bcp.2009.04.011.
5. Liekens, S.; Hernández, A.-I.; Ribatti, D.; De Clercq, E.; Camarasa, M.-J.; Pérez-Pérez, M.-J.; Balzarini, J. The Nucleoside Derivative 5′-O-Trityl-Inosine (KIN59) Suppresses Thymidine Phosphorylase-Triggered Angiogenesis via a Noncompetitive Mechanism of Action*. *Journal of Biological Chemistry* **2004**, *279*, 29598–29605, doi:10.1074/jbc.M402602200.
